# Micronutrients encapsulation in enhanced nanoliposomal carriers by a novel preparative technology

**DOI:** 10.1039/c9ra03022k

**Published:** 2019-06-25

**Authors:** Annalisa Dalmoro, Sabrina Bochicchio, Gaetano Lamberti, Paolo Bertoncin, Barbara Janssens, Anna Angela Barba

**Affiliations:** Eng4Life Srl, Spin-off Accademico Via Fiorentino, 32 83100 Avellino Italy; Dipartimento di Farmacia, Università degli Studi di Salerno via Giovanni Paolo II, Fisciano 132 84084 SA Italy aabarba@unisa.it; Dipartimento di Ingegneria Industriale, Università degli Studi di Salerno via Giovanni Paolo II Fisciano, 132 84084 SA Italy; Dipartimento di Scienze della Vita – Centro Microscopia Elettronica, Università degli Studi di Trieste Via Fleming 31, A/B 34127 Trieste Italy; Department of Pharmaceutics, University of Gent Sint-Pietersnieuwstraat 25 9000 Belgium

## Abstract

Micronutrients administration by fortification of staple and complementary foods is a followed strategy to fight malnutrition and micronutrient deficiencies and related pathologies. There is a great industrial interest in preparation of formulations for joint administration of vitamin D3 and vitamin K2 for providing bone support, promoting heart health and helping boost immunity. To respond to this topic, in this work, uncoated nanoliposomes loaded with vitamin D3 and K2 were successfully prepared, by using a novel, high-yield and semi continuous technique based on simil-microfluidic principles. By the same technique, to promote and to enhance mucoadhesiveness and stability of the produced liposomal structures, chitosan was tested as covering material. By this way polymer–lipid hybrid nanoparticles, encapsulating vitamin D3 and vitamin K2, with improved features in terms of stability, loading and mucoadhesiveness were produced for potential nutraceutical and pharmaceutical applications.

## Abstract 2

Micronutrients administration by liposomal vectors is a growing strategy in fortification processes of staple and complementary foods to fight malnutrition and micronutrient deficiencies and related pathologies.

## Graphical abstract

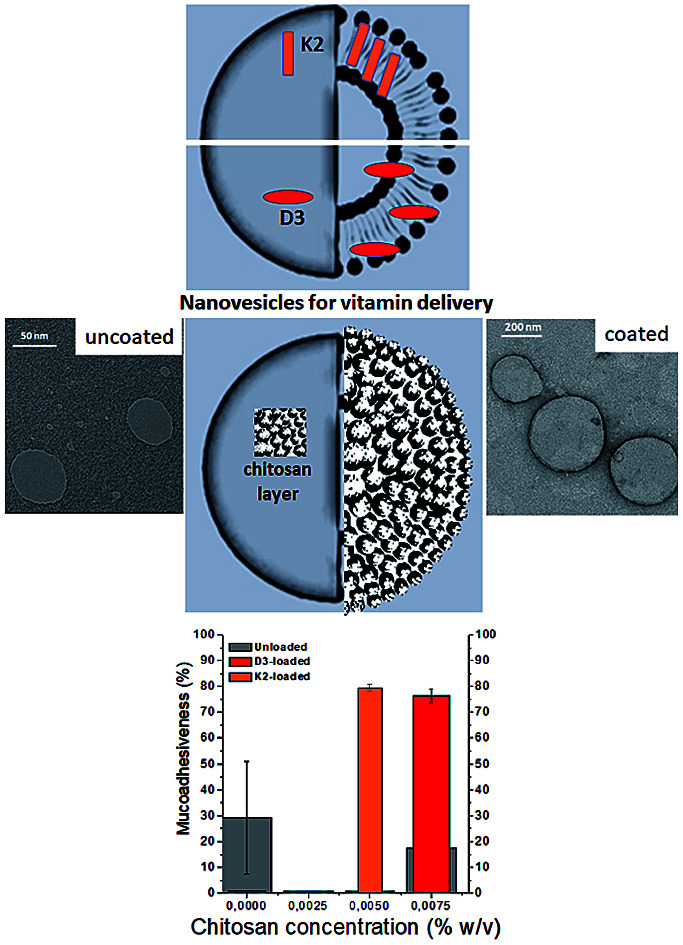

## 1. Introduction

It is proven by numerous researches that micronutrients intake is related to long term health, cognition, healthy development and aging. Health care investigations have shown that inadequate micronutrient status is an issue in industrialized countries as well as in low-income countries where billions of people still suffer from malnutrition and micronutrient deficiencies.^1^ To prevent and/or treat micronutrients deficiency several strategies are currently adopted, such as fortification of staple and complementary foods, provision of supplements.^2^ For these latter purposes encapsulation of micronutrients is, under production point of view, the main approach to ensure suitable dosing, loaded molecules stability and bioavailability.

Vitamins are a class of micronutrients that play a significant role in human growth. The major part cannot be synthetized in the human body or is formed in very little amount. Hence, the importance to provide vitamins in adequate quantities through a diet of fortified food and/or supplements suitably produced by delivery systems.^3,4^ Indeed, in their naked form, vitamins are highly susceptible to degradation and possess poor bioavailability, thus it is essential to wrap vitamins in protective materials in order to prevent their deterioration during both food processes and their uptake in the organism,^5^*i.e.* to enhance their solubility, stability and targeting profile.^6^ The main methods used for encapsulating vitamins are emulsions, solid–lipid nanoparticles, surfactant systems and polymer/lipid encapsulation. The latter includes liposome encapsulation which has recently drawn great interest due to its ability of prolonging shelf life and improving the bioavailability of vitamins and of a wide variety of hydrophilic and hydrophobic molecules such as peptides, proteins, Nucleic Acid Based Drugs (NABDs), which are useful for pharmaceutical, cosmetic, biochemical and nutraceutical purposes.^7–10^ Liposomes are closed vesicular structures constituted by one or more phospholipid bilayers surrounding an aqueous core. They are highly biocompatible and biodegradable drug delivery systems, which, due to their low intrinsic toxicity and immunogenicity and their capability to incorporate hydrophilic and hydrophobic drugs, are the ideal candidates in the controlled release of many kinds of active ingredients. The similarity of their structure to that of cell membranes helps the penetration and overcoming of biological barriers to cellular and tissue uptake of entrapped nutrients.^11^ Despite their several advantages, sometimes they need further stabilization to avoid degradation or aggregation into biological fluids as well as in stocking conditions.

Polymer coating is a promising way to modify the surface properties of liposomes in order to improve their applicability.^12–14^ Among useful polymers, chitosan is a polysaccharide widely used in industry due to its biocompatibility, non-toxic, biodegradable and fungistatic properties, furthermore thanks to its net positive charge it could efficiently interact with negatively charged liposomes due to electrostatic interactions.^15,16^ The coating of liposomes with chitosan has been found to increase their stability, to provide them with mucoadhesive properties, to extend their blood-circulation time, and to decrease the leakage of loaded active principles.^15,17–19^ The point is that the many techniques that have been used so far for an efficacious superficial polymeric coating of nanoliposomes. Currently most applied methods from industries or studied at research level, operate discontinuously on small volumes and require long preparation times. These methodologies can present drastic conditions such as high/low temperatures and pressures or the use of organic solvents that could remain in trace amounts in the final products. For example, in the work of^12^ nanoliposomes containing vitamin E were prepared by organic solvent evaporation technique followed by sonication. Subsequently, for the coating process, a chitosan solution was added under stirring condition to obtain chitosan-coated nano-size liposomes. Besides being a slow and discontinuous process, by means of this bulk techniques it is not possible to obtain a control on the liposomes covering process, thus particles are often characterized by a non-uniform polymeric surface.^12^ The chitosan layer distributed unevenly on the surface of the vesicles leads to a greater propensity to aggregate with the consequent loss of stability. Moreover, the surface portion of the liposomes that is not covered is more subject to degradation with a reduced retention time of the active molecule encapsulated in biological fluids or in storage conditions. Mady and Darwish^20^ used similar conditions, *i.e.* organic solvent evaporation followed by a dropwise chitosan solution – discontinuous bulk techniques, in order to prove that appropriate combinations of the liposomal and chitosan characteristics may produce stable liposomes with specific features. Shin and collaborators used the ethanol injection method for liposomes production, which, although is a rapid process, has the limits imposed by the syringe device volume that make it, once again, a discontinuous technique providing the continuous handling of the prepared suspensions. Finally, the drop-wise chitosan covering method was used in same work^16^ with the same disadvantages seen before.

In this work attention is focused on lipophilic vitamins K and D encapsulation to produce, with a new manufacturing approach characterized by massive and continuous production, stable nanoliposomal chitosan-covered additives as micronutrients delivery model for nutraceutical purposes.

Vitamin D, and in particular the D3 form or cholecalciferol, is able to adjust the *in vivo* metabolization of calcium and phosphorus against osteoporosis, and its deficiency provokes an increased risk of many diseases, such as osteoporosis, some cancers, type 1 diabetes, cardiovascular diseases.^21^ Vitamin K is required by body for both synthesizing some proteins which are prerequisites for blood coagulation and controlling binding of calcium in bones and other tissues, thus a poor vitamin K status is associated with an increased risk of osteoporotic bone fractures.^22^ The most widely used vitamin K form for supplementation is vitamin K2 and more specifically menaquinone-4, more used in trials with bone outcomes, and menaquinone-7, more used in trials with cardiovascular outcomes.^23^ Current evidence reveals that joint supplementation of vitamins D3 and K2 might be more effective than the consumption of either alone in supporting healthy absorption of calcium by D3 action and directing calcium to the bones by K2 action, preventing it from depositing on the arteries and joints. Thus, vitamin D3 and K2 combination provides bone support, promotes heart health and helps boost immunity.^23–25^

Few investigations about vitamin D3 encapsulation in nano systems were found in literature: nanoparticles of whey protein isolate,^26^ nanoparticles of polymers, such as polylactic acid,^27^ solid lipid nanoparticles,^28^ liposomes.^5,29–31^ Vitamin K2 encapsulation was found only in very few patents,^32^ but there was no trace in literature about K2 encapsulation in liposomes. On the contrary, from the industrial view point there are several commercial products containing vitamin D3 and K2 combinations based on liposomal technology (see as examples: Nanonutra, CureSupport, SANUS-q, LipoLife, Doctor's Formulas, QuickSilver Scientific, DesBio, NOW, Protocol, PuraThrive, Clinicians, BioCeuticals, Advanced Therapeutic Medicinals, Actinovo, VitOrtho, Liposol Elivera, Lippomix, LipoPharmacy, GreenLeaves Vitamins, TIB, GoEnergetics, Anatis, Vitasomal, Sanasis – all liposomal products without polymer coatings), attesting the great industrial interest for such a formulation.

Starting from this scenery, this work presents the production of nanoliposomal carriers loaded with D3 and K2 vitamins and coated by chitosan layer to improve features such as load, encapsulation, stability and mucoadhesiveness in order to obtain enhanced nanolipid carriers for vitamin micronutrient delivery. To this aim the novel tested simil-microfluidic technique^33^,^34,35^, characterized by high production yield, continuous regime and mild operative conditions, was adopted both to produce nanoliposomal carriers and to cover the nanostructures with chitosan obtaining, at last, polymer–lipid hybrid delivery systems.

## 2. Experimental

### 2.1 Materials

l-α-Phosphatidylcholine (PC) from soybean, type II-S, 14–23% choline basis (CAS no. 8002-43-5), cholesterol (CHOL) (CAS no. 57-88-5), chitosan (CHIT) with medium molecular weight (190 000–310 000 Da) and 75% degree of deacetylation (DD) (CAS no. 9012-76-4; supplier specification product available on http://www.sigmaaldric.com, cod. 448877), ethanol of analytical grade (CAS no. 64-17-5), glacial acetic acid (CAS no. 64-19-7), vitamin D3 or cholecalciferol (D3) (CAS no. 67-97-0), vitamin K2 (K2) (CAS no. 863-61-6), Triton X-100 (CAS no. 9002-93-1), and mucin from porcine stomach type III, bound sialic acid 0.5–1.5%, partially purified powder (CAS no. 84082-64-4), were purchased from Sigma Aldrich (Milan, Italy).

### 2.2. Methods

#### 2.2.1 Production of uncoated and chitosan-coated liposomal carriers

Liposomal structures and chitosan coated liposomal structures were prepared by the novel simil-microfluidic technique detailed (in terms of plant layout and operative conditions) in our previous works.^34,35^ This technique is based on interdiffusion phenomena occurring between organic/aqueous phases, and is characterized by high production yield, continuous regime and mild operative conditions. Briefly, a lipophilic solution was prepared by weighing 470 mg of phosphatidylcholine and 94 mg of cholesterol and dissolving these amounts in 10 mL of ethanol. 32.4 mg of cholecalciferol, or vitamin K2, were added to the lipophilic solution and magnetically stirred until complete dissolution. 100 mL of deionized water was used as hydration solution. The lipophilic and hydration solutions were put in contact in the simil-microfluidic set-up, keeping the conditions of flow-rate (1/10 lipophilic/hydration solutions) already used before to obtain a hydro-alcoholic solution containing liposomes encapsulating vitamin D3 or vitamin K2. Unloaded liposomes were also prepared for comparison (control). The final suspension was first magnetically stirred for 1 h, then it was split in two aliquots, one subjected to characterization, another to chitosan coverage.

Different chitosan concentrations, *i.e.* 0.0025%, 0.005%, 0.00625%, 0.0075%, 0.01% w/v, in a 0.5% (v/v) acetic acid solution, were tested in order to check the best coverage of unloaded, D3 and K2 liposomes. Briefly, the previously prepared suspension of nanometric unilamellar liposomes and the chitosan solution were pushed in the simil-microfluidic set-up at the same flow rate (25 mL min^−1^) to produce a suspension of chitosan-coated liposomes, subjected first to stirring for 1 h, then to characterization.

#### 2.2.2 Particles size and zeta potential analysis

Size measurements of both uncoated and coated liposomes, the dynamic light scattering (DLS) method was chosen by using the Zetasizer Nano ZS (Malvern, UK), which has a noninvasive backscatter (NIBS) optics to define the liposomes average hydrodynamic diameter (size) and the size distribution (PDI). In particular, a detection angle of 173°, able to measure the particles size of concentrated and turbid samples, was used. The numerical size distribution was made up by plotting the number of particles *versus* the particle size, the PDI and *Z*-average values were calculated by the Zetasizer Nano ZS software. The zeta potential (*ζ*) was measured by transferring an aliquot of the sample solution into a capillary cell and by performing the analysis by the Photon Correlation Spectroscopy (PCS) in the Zetasizer Nano ZS instrument. The measurements of each sample (D3-loaded; K2-loaded, and unloaded liposomes) were performed in triplicate and all the results were expressed as average values with the corresponding standard deviations, SD.

#### 2.2.3 Transmission electron microscopy (TEM)

Structural characterization of uncoated and chitosan coated nanoliposomal vesicles was carried out by transmission electron microscopy, TEM (EM 208, Philips), equipped with camera Olympus Quemesa (EMSIS GmbH and Software RADIUS). In brief, samples were produced, before the observation, diluted 1 : 10 v/v with distilled water and were deposited about 10 μL on a carbon support film on copper specimen grid mesh 200 (Electron Microscopy Sciences). Then, after 5 min, samples were negatively stained with 1% w/v of uranyl acetate solution for 5 min, drying the exceeded water, and finally were completely dried at room temperature.

#### 2.2.4 Encapsulation efficiency and loading

The encapsulation efficiency was assessed by lysing the liposomes and analyzing the entrapped vitamin. 3 mL of the sample, containing liposomes encapsulating the vitamin (D3 or K2), were subjected to centrifuge for 60 minutes at 35 000 rpm (118 443 × *g*), under vacuum at 4 °C (to avoid liposomes overheating during centrifugation, used centrifuge: Beckman Optima L-90K centrifuge with SW 55 Ti rotor, Beckman Instruments, Palo Alto, CA, USA), with the aim to separate the supernatant, containing the unencapsulated vitamin, from the precipitated liposomes (pellet). After centrifugation the supernatant was thus removed by aspiration with a Gilson pipette and substituted by a detergent for destroying liposomes and detect the encapsulated vitamin. In particular, for vitamin D3-loaded liposomes, the pellet was treated with 3 mL of ethanol, instead for vitamin K2-loaded ones, the pellet was treated with 3 mL of Triton X-100 at 1% (v/v). Triton X-100 was not used for lysing D3-loaded liposomes because it absorbs in the same range as vitamin D3 and disturbs in this way the UV-VIS quantification. The pellet was left to incubate for approximately 30 minutes and afterwards sonicated for one minute at 100% amplitude (VCX 130 PB Ultrasonic Processors, 130 W, frequency 20 kHz, Sonics & Materials Inc., CT, USA). Both the supernatant and pellet of each sample were submitted to UV-VIS spectrophotometric analysis (Lambda 35, PerkinElmer, Monza, Italy) by investigating an absorption spectrum from 200 nm to 400 nm, and inside it choosing the maximal wavelength of absorbance at 270 nm for vitamin D3 and 329 nm for vitamin K2. The encapsulation efficiency (EE, %) was consequently defined as the percentage ratio between the amount of vitamin (D3 or K2) detected in the pellet, thus encapsulated in liposomes (for both chitosan-coated and uncoated liposomes) and the amount of vitamin initially included in the formulation (*i.e.* inserted in the lipophilic solution), as expressed by the following equation:1
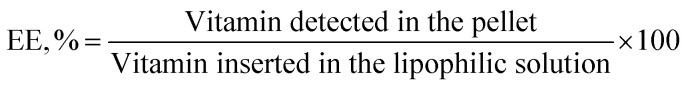


Moreover, the sum of vitamin detected in the pellet, *i.e.* encapsulated in liposomes, and vitamin found in the supernatant, *i.e.* unencapsulated, was compared with the initial amount of vitamin used for the formulation in order to verify that it was not degraded by the process.

Vitamin loading percentage was determined as the percentage ratio between the amount of vitamin encapsulated in liposomes and the total amount of components included in the formulations, *i.e.* PC, CHOL, vitamin (D3 or K2), and CHIT for coated liposomes, calculated using the following equation:2
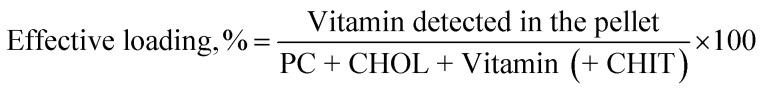


The theoretical loading has as numerator in eqn (2) the vitamin inserted in the lipophilic solution.

All the kinds of measurements were performed in triplicate and all the results were expressed as average values with the corresponding standard deviations, SD.

#### 2.2.5 Mucin binding assay

Mucoadhesive properties of uncoated and chitosan-coated (unloaded, D3-loaded, or K2-loaded) liposomes was evaluated as already described in ref. 34. Briefly, mucin was dissolved in phosphate buffer (pH 7.4) to reach a concentration of 400 μg mL^−1^. Then, 2 mL of mucin solution were mixed with 2 mL of liposomes suspension (1 : 1, v/v) and left to incubate at room temperature (23 °C) for 2 h. Afterwards, 2 mL of the liposome/mucin solution were centrifuged for 60 min at a relative centrifugal force of 118 443 × *g* and 4 °C (Beckman Optima L-90K centrifuge with SW 55 Ti rotor, Beckman Instruments, Palo Alto, CA, USA) in order to remove the supernatant. Mucin not absorbed by the pellet, thus kept free in the supernatant itself, was detected by UV spectrophotometry (Lambda 35, PerkinElmer, Monza, Italy) at 384 nm. The sample mucoadhesiveness was defined as Mucin Binding Efficiency (MB eff., %), calculated as the percentage ratio between the mucin bounded to liposomes, expressed as the difference between the initial mucin concentration, *C*_0_, in the liposome/mucin solution (200 μg mL^−1^), and the free mucin detected in the supernatant, *C*_S_, and the initial mucin concentration *C*_0_, as visible by the following equation:3
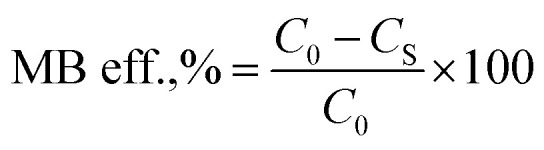


All the determinations were performed in triplicate and the results were expressed as average values with standard deviation, SD.

#### 2.2.6 Turbidimetry for liposome solubilization

Turbidimetry assay was carried out to examine the stability of the liposomes by measuring their Nephelometric Turbidity Units (NTU) by means of a turbidimeter (PCE-TUM 20, PCE Instruments, Italy), upon the continuous addition of Triton X-100, a non-ionic surfactant. The test was performed on the uncoated and chitosan-coated, unloaded, D3 and K2 loaded liposomes. 5 mL of the sample was diluted up to 10 mL using deionized water and transferred into a glass cuvette. The turbidimeter was first calibrated, using a 0 NTU and 100 NTU calibration solution. Then, the turbidity of the sample was measured, each time after a certain amount of Triton X-100 was added. The NTU was noted until the sample was fully solubilized. Between every measurement, the sample solution was magnetically stirred in order to obtain a homogeneous distribution and solubilisation of Triton X-100. All the measurements were performed in triplicate, the results were expressed as average values with standard deviation SD, and plotted against the Triton X-100 concentration% (v/v) in the solution.

#### 2.2.7 Stability during storage

In order to investigate the stability of the produced liposomal carriers in function of time, 20 mL of the uncoated and chitosan-coated, D3 and K2 loaded samples were sealed and stored at 4–6 °C during 1 month. Afterwards, the samples were re-analysed for size, PDI, zeta potential, encapsulation efficiency and mucoadhesive properties. Moreover, structural characteristics were also observed by TEM investigation.

#### 2.2.8 Statistical evaluation

The student *t*-test was carried out for the comparison of two mean values with their associated standard deviations. A level of significance of 5% was considered acceptable. Excel data sheet was used to manage experimental achieved values.

## 3. Results

### 3.1 Properties of uncoated nanoliposomes

As expected, lipid unilamellar vesicles produced by the simil-microfluidic technique have shown nanodimensions with a reduced PDI (Table 1). D3-loaded nanoliposomes had size properties similar to unloaded ones, unlike K2-loaded nanoliposomes. As visible in Table 1, numerical size and *Z*-average were about 90 nm and 250 nm, respectively, for both unloaded and D3-loaded nanoliposomes. On the contrary, starting from roughly 90 nm in size for unloaded nanoliposomes, size and PDI values for K2-loaded nanoliposomes were higher, 150 nm and 290 nm, respectively. Looking at the differences in structure of vitamin D3 (MW 384.33 amu) and vitamin K2 (MW 444.7 amu), the aliphatic side chain of isoprenoid residues gives to vitamin K2 a more stretched structure whereas vitamin D3 is more compact. This could lead to a different incorporation of vitamin K2 in the nanoliposome's lipid bilayer. In effect, according to ref. 29, vitamin D3 into the lipid bilayer is able to interchange with phospholipid membrane, causing disordered structures, lower melting temperatures and decrease in the membrane fluidity,^36^ compared to that of unloaded liposomes.^30^ found that vitamin D3 is incorporated into the phosphatidylcholine bilayer and intercalated between the hydrocarbon chains of phospholipid molecules, thereby disturbing the gel–liquid crystalline phase transition.^37^ proved that vitamin K1 (with a similar structure to vitamin K2), in opposition to D3 behaviour, had a limited miscibility with phosphatidylcholine. In particular, the methyl substituents of the phytanoyl chain of K1 prevent its accommodation to the all-*trans* configurations adopted by the chains of phosphatidylcholine, thus the incorporation of K1 into the gel phase is thermodynamically unfavourable. In effect, at high K1 concentrations, new phase rich in K1 was detected.

**Table 1:** Encapsulation efficiency, numerical size, *Z*-average, PDI and zeta potential of uncoated unloaded, D3-loaded, K2-loaded nanoliposomes

Properties	Unloaded nanoliposomes	D3-loaded nanoliposomes	K2-loaded nanoliposomes
EE% ± SD	—	88.4 ± 2.5	94.7 ± 0.8
Numerical size (nm) ± SD	88.3 ± 19.0	87.4 ± 17.3	145 ± 32.7
*Z*-Average (nm) ± SD	252.8 ± 2.1	247.8 ± 1.2	289.0 ± 5.6
PDI ± SD	0.38 ± 0.02	0.40 ± 0.07	0.32 ± 0.02
Zeta potential (mV) ± SD	−35.4 ± 0.8	−38.5 ± 1.6	−36.2 ± 0.3

**Fig. 1:**
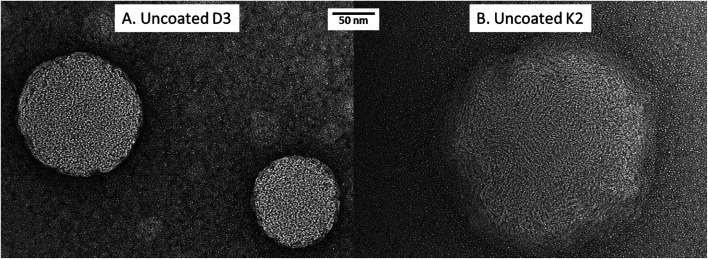
TEM images of uncoated D3 (A) and K2 (B) loaded nanoliposomes.

This different structure between vitamin D3 and vitamin K2 loaded nanoliposomes was also visible in TEM pictures (Fig. 1A and B): larger nanoparticles and a more irregular structure were observed for K2-loaded nanoliposomes (Fig. 1B) than D3 ones (Fig. 1A).

For both vitamin D3 and vitamin K2-loaded nanoliposomes, preliminary experiments proved that increasing the vitamin concentration up to 590 μg mL^−1^ led to much more acceptable values regarding the encapsulation efficiency. In particular, EE was of 88.4% and 94.7%, for vitamin D3 and vitamin K2, respectively (Table 1), values comparable to those reported for similar systems (about 95% for K1-loaded liposomes in ref. 37, and 86% for D3-loaded liposomes in ref. 28). However, increasing the vitamin concentrations to even higher levels did not brought to higher EEs.^28^ At some point, the increase in vitamin concentration lead to a decrease in EE due to reaching the maximum encapsulation ability of the liposomal structures. Concentrations added beyond the encapsulation ability of the liposomes resulted in free, unencapsulated vitamin molecules.^28^ Moreover, the fact that the K2 encapsulation was higher than D3 was probably due to the more hydrophobic character of K2 (water solubility in mg l^−1^: 2.70 × 10^−7^ for K2, 2.22 × 10^−5^ for D3).

### 3.2 Chitosan coverage of liposomes

As described in Section 2.2.1, the unloaded, vitamin D3 and vitamin K2 loaded nanoliposomes were coated by using different concentrations of chitosan, varying from 0.0025% to 0.01% (higher concentrations gave aggregation phenomena). For the unloaded and vitamin D3-loaded nanoliposomes, no problems were detected during the coating process. However, the coating of vitamin K2-loaded nanoliposomes, performed with concentrations higher than 0.005% of chitosan, showed visual aggregation, and were thus not suited to work with. The mechanism behind the aggregation of the particles is strongly associated with the concentration of chitosan added to the liposomal suspension. In literature is found that, in order to achieve stable and fully coated liposomes, a chitosan concentration within a specific range must be added to the system. This range reaches from a minimum concentration to the saturation concentration, required to fully cover the liposome's surface. Any excess amount of chitosan, which is not adsorbed on the liposome's surface, will generate an attractive force promoting the aggregation of the particles.^34,38^ The fact that this aggregation did not occur with the unloaded and vitamin D3 nanoliposomes, suggests that vitamin K2 might interact in a different way with chitosan on the surface of the vesicle, due to the previously detailed differences in structure between vitamin D3 and vitamin K2, which lead not only to a different incorporation of vitamin K2 in the liposome's lipid bilayer, but also induce interaction with chitosan causing a different coverage of the surface.

#### 3.2.1 Size and zeta potential

**Fig. 2:**
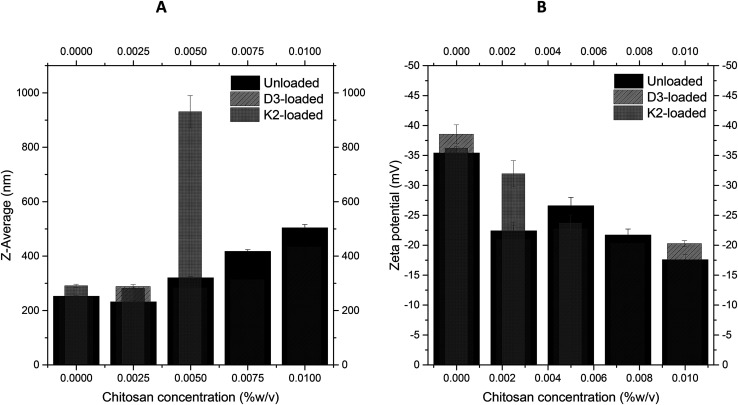
Variation of *Z*-average (A) and *Z*-potential (B) of unloaded, D3-loaded, and K2-loaded nanoliposomes at increasing chitosan solution concentrations.

As described before, numerical size, *Z*-average and polydispersity index (PDI) are essential features influencing the stability, solubility, release rate and bioavailability of the liposomes.^28^ The particle size influences the stability of the liposomal suspension, and also the release rate. As the particle size increases, the surface-to-volume ratio decreases and the dissociation occurs more slowly.^4^Table 2 and Fig. 2A and B show the results for the analysis of size (numerical and *Z*-average), PDI and zeta potential for unloaded liposomes (without vitamins), vitamin D3-loaded, vitamin K2-loaded, respectively, coated with the different chitosan concentrations. For unloaded liposomes (Table 2), the comparison among the numerical sizes obtained for the different coatings shows that the size of liposomes coated with 0.01% (w/v) of chitosan was significantly (*P* < 0.05) higher than the size of liposomes coated with lower chitosan concentrations: the numerical size of liposomes from 0 to 0.0075% (w/v) chitosan were considered similar (*P* > 0.05). *Z*-Average values (Fig. 2A) increased from about 250 nm without coating up to 500 nm with 0.01% w/v of chitosan coating, demonstrating that the upper chitosan concentration was the most successful for a good coverage. Higher concentrations than 0.01% w/v were not used because they were proved to be cause of flocculation of coated nanoliposomes as reported in a previous work.^35^ Similar results were found for vitamin-D3-loaded nanoliposomes with some differences. In particular, the numerical size of the uncoated liposomes was significantly lower than the sizes at higher chitosan concentrations, together with a higher PDI (data not showed). The numerical sizes from 0.0025 to 0.0075% coated liposomes were considered similar (*P* > 0.05), and no significant change in PDI was detected. This result creates the hypothesis that some coverage took place at a chitosan concentration starting from 0.0025%. However, the *Z*-average of 0.01% (w/v) coated nanoliposomes had a significantly higher value (*P* < 0.05) compared to the liposomes coated with lower chitosan concentrations, indicating a more successful coverage of the liposome's surface. K2-loaded nanoliposomes showed no significant difference in numerical size (data not showed), *Z*-average and PDI (data not showed) for the 0% (w/v) and 0.0025% (w/v) chitosan concentrations, suggesting no coverage up to this concentration. On the contrary, a larger numerical size of 278 ± 105 nm, PDI of 0.71 ± 0.085 and *Z*-average of 930 ± 59.5 nm was obtained for 0.005% (w/v) chitosan-coated nanoliposomes. As previously accounted, vitamin K2 stretched structure might provoke a different way of incorporation in the nanoliposome's surface and its higher presence in the outer layer of the liposome's membrane. This causes an uneven coverage with chitosan which could be reflected in the high values of *Z*-average and PDI. The variation of *Z*-average by increasing the chitosan concentration for the three different kinds of samples (unloaded, D3-loaded, K2-loaded nanoliposomes) is more clear in Fig. 2A, where it is evident the similar behaviour between D3-loaded nanoliposomes and unloaded ones, and the great difference for K2-loaded nanoliposomes. As regards the variation of *Z*-potential in function of the chitosan concentration, it is evident from Fig. 2B that as the chitosan concentration increases, the zeta potential becomes less negative and eventually tends to reach a more or less constant value, for unloaded, D3-loaded and K2-loaded liposomes, due to the interaction between the cationic chitosan and the negatively charged lipid bilayer. An explanation for the striving to constant value could be that the chitosan fully covered the liposome's membrane at some point, allowing no further adsorption. In particular, the fact that also coated nanoliposomes remain negatively charged is an advantage. In fact, as described by,^39^ a layer of negatively charged polysaccharide improves the stability avoiding aggregation phenomena. This and the above described results involving the particle size confirm the successful coverage of the nanoliposomes' surface at a chitosan concentration of 0.01% (w/v) for unload and D3-loaded nanoliposomes and the 0.005% (w/v) for K2-loaded nanoliposomes.

**Table 2:** Numerical size, *Z*-average, PDI and zeta potential of unloaded nanoliposomes, coated by using different concentrations of chitosan solutions

Chitosan concentration (% w/v)	Numerical size (nm) ± SD	*Z*-Average (nm) ± SD	PDI ± SD	Zeta potential (mV) ± SD
0	88.3 ± 19.0	252.8 ± 2.1	0.38 ± 0.02	−35.4 ± 0.8
0.0025	85.8 ± 39.6	232.2 ± 4.8	0.29 ± 0.02	−22.4 ± 0.9
0.005	114 ± 32.3	320.6 ± 4.2	0.36 ± 0.05	−26.6 ± 1.4
0.00625	94.6 ± 35.7	322.5 ± 2.2	0.28 ± 0.07	−18.7 ± 2.0
0.0075	100 ± 30.8	417.4 ± 6.2	0.29 ± 0.02	−21.7 ± 1.0
0.01	257 ± 37.6	504.4 ± 12.1	0.34 ± 0.03	−17.6 ± 0.8

#### 3.2.2 Mucoadhesive properties

**Fig. 3:**
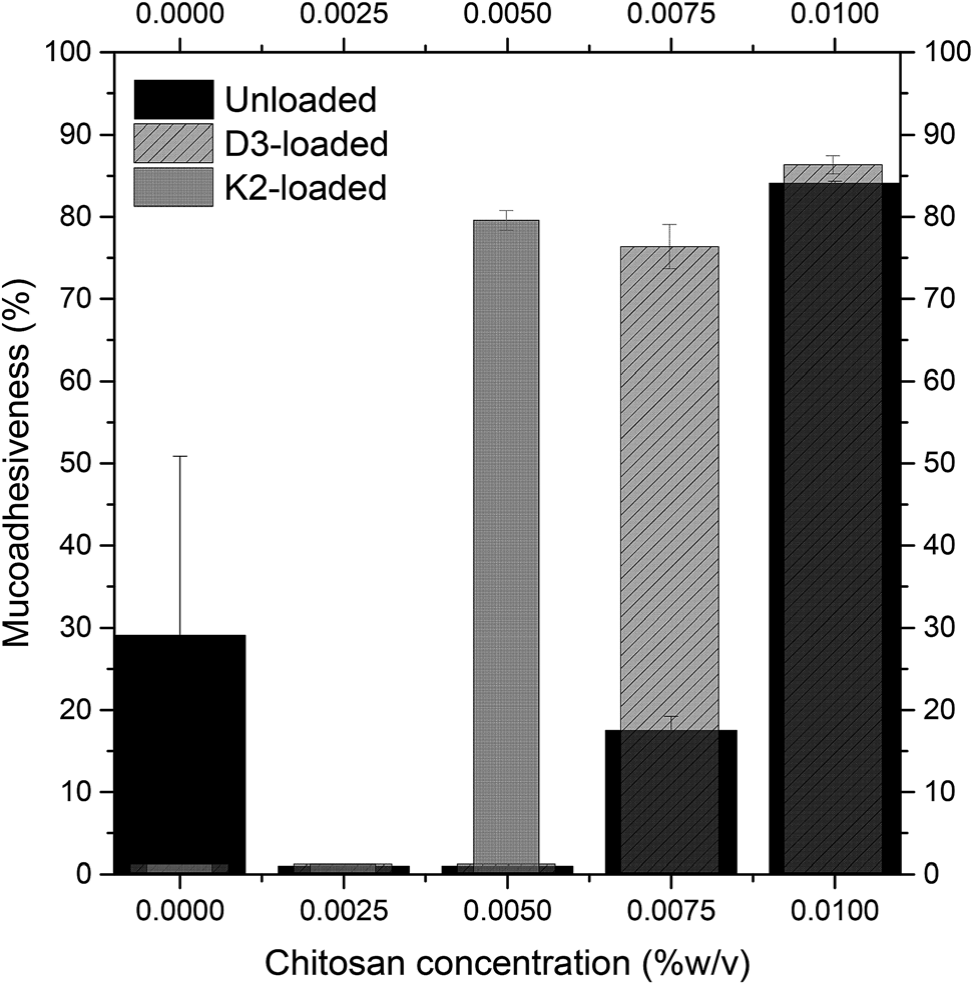
Variation of mucoadhesiveness of unloaded (black), D3-loaded (diagonal lines filling), and K2-loaded (dark gray) nanoliposomes at increasing chitosan concentrations.

Although the results for size and zeta potential confirm the successful coverage of the nanoliposomes' surface, it is still important to determine the mucoadhesive properties in order to obtain confirmation of the bioadhesiveness of the produced vitamin delivery systems. From Fig. 3 it can be seen that for both unloaded and D3-loaded nanoliposomes, as expected, the highest mucoadhesiveness of about 85% was obtained in nanoliposomes coated with 0.01% (w/v) of chitosan. With a chitosan concentration of 0.0075% (w/v), D3-loaded nanoliposomes presented higher mucoadhesive properties with respect to unloaded ones. Concentrations under 0.0075% presented absence of mucoadhesiveness, probably due to the fact that chitosan coverage is very limited, thus not sufficient to assure adhesion of particles to mucin. These results agree with literature ones where not covered particles are known to be not mucoadhesive.^40,41^ Vitamin K2-loaded nanoliposomes had no mucoadhesiveness up to the 0.005% (w/v) of chitosan, where they showed a fairly high mucoadhesiveness of about 80%. Results from mucin binding assay are in line with results about size and *Z* potential, confirming the best coverage with 0.01% w/v for unloaded and D3-loaded nanoliposomes, and with 0.005% per K2-loaded nanoliposomes.

#### 3.2.3 Turbidimetry

**Fig. 4:**
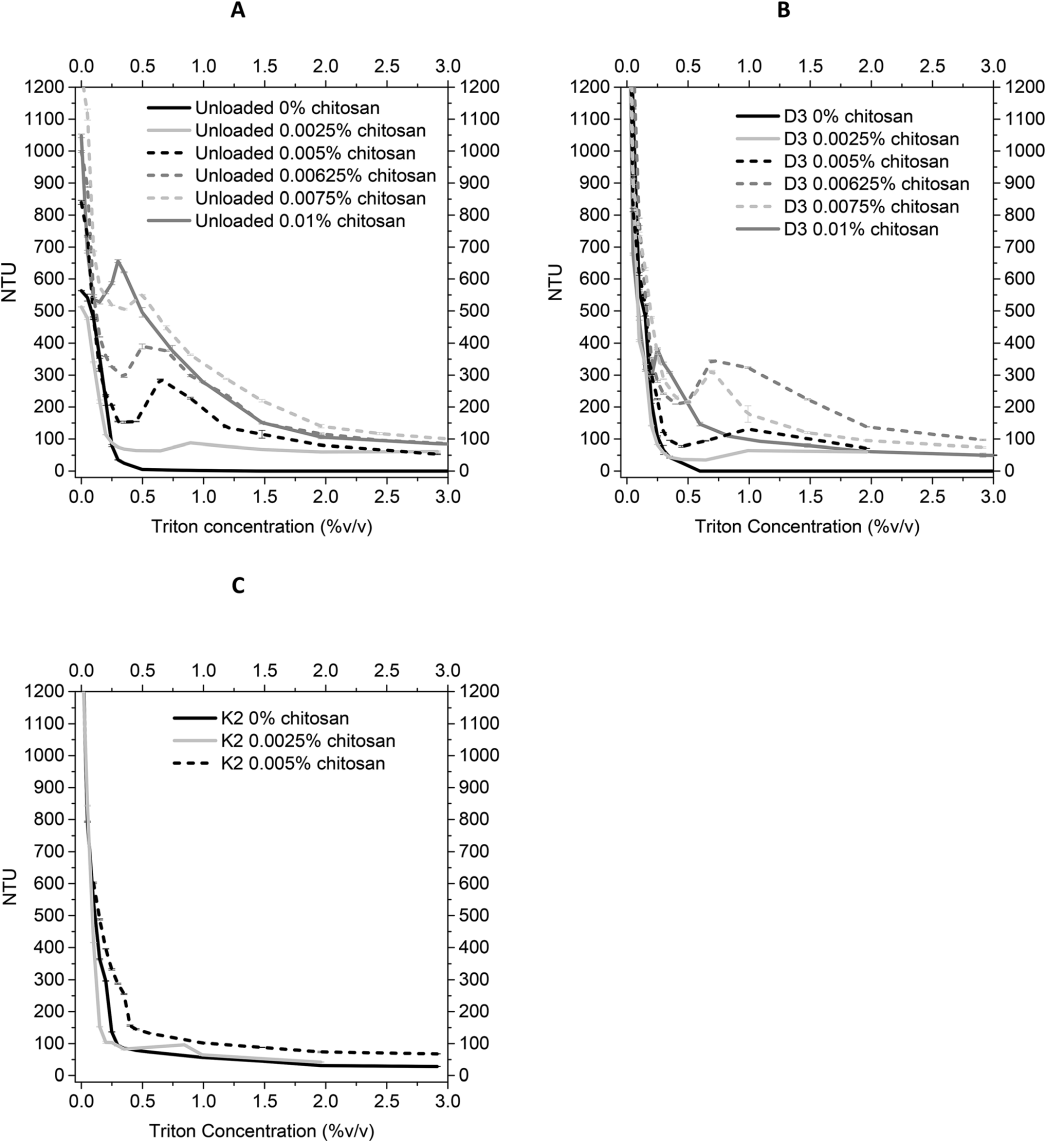
Stability of unloaded (A), D3-loaded (B), and K2-loaded (C) nanoliposomes at increasing chitosan concentrations, evaluated by turbidimetry measurements of samples suspensions after detergent Triton X-100 adding.

The solubilization of uncoatead and coated produced nanostructures, by the increasing addition of the non-ionic detergent Triton X-100, was performed to determine the stability of the chitosan–lipid interaction. Besides determining the stability, the turbidimetric methods provides an extra confirmation on the coverage of the liposome's surface. From Fig. 4 it can be noted that increasing concentrations of Triton X-100 lead to solubilize all the three typologies of nanoliposomes analysed (decreasing NTU values). Comparing the uncoated liposomes to the coated ones, it can be seen that for all amounts of Triton X-100 added, the uncoated liposomes showed a lower turbidity, thus less stability, than the chitosan coated ones, as already seen in literature.^42^ The different prepared samples had also different solubilisation profiles. In particular, uncoated nanoliposomes showed a continuously decreasing NTU curve, attesting a constant insertion of detergent monomers into the phospholipid bilayer with its consequent immediate solubilisation. Instead coated liposomes were characterized by a first stage of decreasing NTU, then a sharp increase causing a peak, due to incorporation of detergent molecules in the chitosan coating layer before reaching the phospholipid bilayer, and a final decrease up to the minimal value, as already seen in other research works.^43^ It can be noted that the peak, due to the interference of chitosan layer, moved at higher NTU values and lower Triton X-100 concentrations passing from lower to higher chitosan concentrations. This confirms that the higher the chitosan concentration, thus the more surface is covered, the more resistant is the system. For example, for unloaded liposomes (Fig. 4A), the profile of 0.0025% w/v coated nanoliposomes attests that the surface is only slightly covered because it was characterized by an initial sharp decrease in NTU (due to solubilisation of lipid membrane), and a consequent little peak (about 100 NTU, demonstrating the presence of a little amount of chitosan on the surface) at high concentration of Triton X-100. On the contrary, it was confirmed that the 0.1% w/v of chitosan assured a full coverage of nanoliposomes' surface, due to the presence of a high peak (of about 650 NTU) at very low Triton X-100 concentrations. The same trend was also observed for D3-loaded liposomes (Fig. 4B), but the peak values, for each degree of coverage, were at NTU values lower than the corresponding ones for unloaded liposomes, confirming the perturbation of liposome structure by D3 insertion, as previously said.^29^ The larger interaction between K2 and the phospholipid bilayer was also evident in NTU profiles (Fig. 4C). In effect the 0.0025% (w/v) coated nanoliposomes showed a similar profile to the uncoated ones, confirming the failure of coverage. On the contrary, the 0.005% (w/v) profile showed a fairly different behaviour from the uncoated nanoliposomes, indicating that the coverage actually took place.

### 3.3 Properties of polymer–lipid hybrid delivery system

From previous results about the behaviour of size and zeta potential, mucoadhesive properties, and the turbidimetry measurements of the lipid hybrid delivery systems suspensions in function of the chitosan coating degree, 0.01% w/v and 0.005% w/v chitosan concentrations were chosen for covering D3-loaded and K2-loaded nanoliposomes, respectively.

#### 3.3.1 Encapsulation efficiency

The encapsulation efficiency and effective loading results for both vitamin D3 and vitamin K2 chitosan-coated nanoliposomes (together with the uncoated ones) are resumed in Table 3. In both cases the entrapment efficiency was clearly positively affected by the chitosan coating, as it increased from 88% to 98% for D3-loaded nanoliposomes and from 95% to 98% for K2-loaded ones. This is reasonably due to the fact that during the coating process, chitosan covers the surface of the liposomes and fill the gaps in phospholipid bilayer thought instauration of various forces such as van der Waals, hydrogen bonding and electrostatic interactions.^15,44,45^ The effective loading was of about 10% for all the formulations.

**Table 3:** Encapsulation efficiency for vitamin D3 and vitamin K2 uncoated and chitosan-coated nanoliposomes

	D3 nanoliposomes	D3 chitosan-coated nanoliposomes (0.01% w/v)	K2 nanoliposomes	K2 chitosan-coated nanoliposomes (0.005% w/v)
EE (%) ± SD	88.4 ± 2.5	98.3 ± 0.5	94.7 ± 0.8	98.2 ± 0.6
Theoretical loading (%)	10.4	10.2	10.4	10.3
Effective loading (%)	9.2 ± 0.3	10.0 ± 0.0	9.8 ± 0.1	10.1 ± 0.1

#### 3.3.2 Morphology and stability during storage

**Fig. 5:**
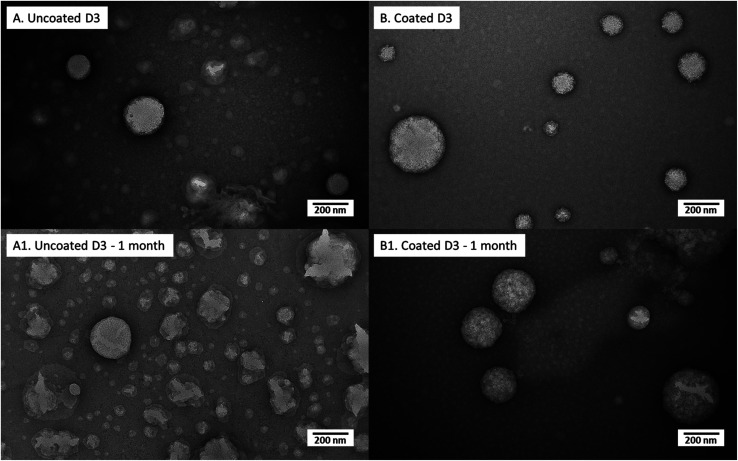
Transmission electron micrographs of uncoated D3 nanoliposomes and coated D3 nanoliposomes suddenly after preparation (A and B, respectively) and after 1 month of storage (A1 and B1, respectively).

**Fig. 6:**
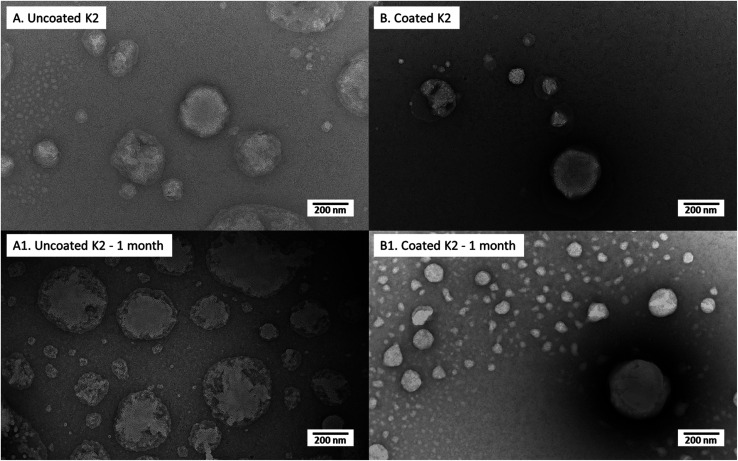
Transmission electron micrographs of uncoated K2 nanoliposomes and coated K2 nanoliposomes suddenly after preparation (A and B, respectively) and after 1 month of storage (A1 and B1, respectively).

Morphology investigation of both D3 and K2 loaded nanoliposomes in uncoated and coated forms, right after preparation and after 1 month of storage are shown in TEM pictures of Fig. 5 (for D3-loaded nanoliposomes) and Fig. 6 (for K2-loaded nanoliposomes). In general no aggregation among nanoliposomal structures (both uncoated and chitosan-coated) was observed, in agreement with the results obtained by,^39^ demonstrating that negatively charged polysaccharide coated liposomes had more stability against aggregation.

D3-loaded nanoliposomes kept intact their structure also after chitosan coverage (Fig. 5); in effect both uncoated (Fig. 5A) and coated ones (Fig. 5B) showed a spherical shape. Moreover, the chitosan layer surrounding liposomes, clearly visualized in TEM photos of a previous work^35^ in unloaded liposomes, here was not evident perhaps for the perturbation of liposomal structure by D3 insertion, causing a thinner coating thickness (not visible in photo), which could be the reason of the lower stability (with respect to unloaded liposomes) during the previously discussed turbidimetry tests. After 1 month of storage, the uncoated nanoliposomes (Fig. 6A1) showed signs of some disaggregation of the external layer, as confirmed by the increment of *Z*-average size (from 344 nm at time 0 to 503 nm after 1 month) and of PDI (Table 4). However, it was only a superficial effect because *Z*-potential was kept unchanged, as well as the encapsulation efficiency (stable at around 88%). Looking at the 0.01% (w/v) coated D3 nanoliposomes stored for 1 month in Fig. 6B1, it is evident that their morphology seems unchanged with respect to freshly prepared liposomes. This behaviour is confirmed by all other properties, *i.e.* numerical size, *Z*-average, *Z*-potential, PDI, encapsulation efficiency and mucoadhesiveness, which remained unchanged (*P* > 0.05) (Table 4). Thus, also in storage conditions, the chitosan coverage confirmed to impart stability to liposomes, in agreement with previous studies demonstrating that polymers forming a layer around liposomes reduced the oxidation of the lipids and prevented the leakage of drugs.^46,47^

Properties of D3/K2 loaded, uncoated and 0.01% w/v chitosan-coated nanoliposomes, suddenly after preparation (*t* = 0) and after 1 month of storage (*t* = 1)

**Table:** 

	Uncoated D3-nanoliposomes		Chitosan-coated D3-nanoliposomes	
Time	*t* = 0	*t* = 1	*t* = 0	*t* = 1
Size (nm) ± SD	87.4 ± 17.3	117.0 ± 42.6	235.1 ± 29.3	211.1 ± 76.6
*Z*-Average (nm) ±S D	344.0 ± 61.9	503.0 ± 10.9	434.0 ± 31.5	393.9 ± 11.7
PDI ± SD	0.40 ± 0.07	0.50 ± 0.03	0.25 ± 0.05	0.29 ± 0.04
Zeta potential (mV) ± SD	−38.5 ± 1.6	−37.2 ± 2.1	−20.2 ± 0.5	−18.9 ± 1.5
EE (%) ± SD	88.4 ± 2.5	87.3 ± 0.7	98.3 ± 0.5	98.4 ± 0.1
Mucoadhesivity ± SD	0.0 ± 0.0	3.2 ± 7.7	86.3 ± 1.1	88.5 ± 3.5

**Table:** 

	Uncoated K2-nanoliposomes		Chitosan-coated K2-nanoliposomes	
Time	*t* = 0	*t* = 1	*t* = 0	*t* = 1
Size (nm) ± SD	144.8 ± 32.7	95.6 ± 33.0	278.5 ± 105.0	324.7 ± 122.0
*Z*-Average (nm) ± SD	289.1 ± 5.6	322.9 ± 6.85	930.1 ± 59.5	726.2 ± 35.0
PDI ± SD	0.31 ± 0.02	0.37 ± 0.03	0.71 ± 0.09	0.50 ± 0.09
Zeta potential (mV) ± SD	−36.2 ± 0.3	−36.6 ± 3.0	−23.7 ± 1.3	−21.1 ± 0.6
EE (%) ± SD	94.7 ± 0.8	93.8 ± 0.4	98.2 ± 0.6	95.2 ± 0.2
Mucoadhesivity ± SD	0.0 ± 0.0	4.0 ± 5.4	79.5 ± 1.2	77.8 ± 0.4

Similarly, to D3-loaded nanoliposomes, the morphology (Fig. 6) and properties (Table 4) of uncoated and 0.005% (w/v) coated vitamin K2 nanoliposomes right after preparation and after 1 month of storage were compared. The TEM images of just produced samples (Fig. 6A) showed spherical particles with some superficial defects due to vitamin K2 presence. The behaviour of uncoated K2-loaded nanoliposomes after 1 month of storage showed no significant change in number size, but an increase in *Z*-average and PDI (*P* < 0.05) (Table 4), confirmed by a kind of swelling of liposome matrix visible in TEM image (Fig. 6A1). However, the zeta potential was not affected, together with the encapsulation efficiency and the mucoadhesiveness, which both remained unchanged (*P* > 0.05) (Table 4). The 0.005% (w/v) chitosan coated K2 nanoliposomes showed irregular coating of liposomes surface with more aggregates (Fig. 6B), explaining the high *Z*-average (930 nm) and PDI (0.7) values (Table 4). The storage for 1 month did not cause change in number size over time (*P* > 0.05), as well as for the encapsulation efficiency and the mucoadhesiveness. However, the *Z*-average and PDI decreased, as well as a little bit the zeta potential (*P* < 0.05) (Table 4). This is an indication of the stabilization of the suspension over time, becoming more monodisperse, as confirmed by TEM image (Fig. 6B1), which shows more regular structures compared to the freshly prepared sample (Fig. 6B). Moreover, by comparing images of uncoated (Fig. 6A1) and coated (Fig. 6B1) K2-loaded nanoliposomes, appearing uneven and compact, respectively, it is evident that also in this case chitosan acts as protection against degradation over time.

## 4. Conclusions

Nanoliposomal carriers and chitosan coated nanoliposomes, encapsulating vitamin D3 and vitamin K2, were both successfully produced by the simil-microfluidic technique, with the advantages of massive production, operating at environmental conditions and continuously.

Investigation on uncoated nanoliposomes showed high encapsulation efficiencies, especially for vitamin K2 (EE: 95%) due to its more hydrophobic character. Uncoated K2 an D3 loaded nanoliposomes have been shown poor mucoadhesive characteristics thus chitosan coating was performed to overcome this issue. The coverage efficacy was proven to be dependent on chitosan concentrations and on kind of enwrapped vitamin in the liposomal structure. In this study, the best coverage was obtained with 0.01% w/v chitosan for unloaded and D3-loaded liposomes, and with 0.005% per K2-loaded liposomes. Moreover, the best chitosan coverage for each liposomal formulation has led an increase of the entrapment efficiency from 88% to 98% for D3-loaded liposomes and from 95% to 98% for K2-loaded ones. This enhancement is occurred, reasonably, due to the fact that during the coating process, chitosan covers the surface of the liposomes and fills the gaps in the hydrophobic bilayer.

After 1 month of storage, the uncoated nanoliposomes (both of D3 and K2) showed signs of some disaggregation of the external layer (as confirmed by TEM photographs and by increments of *Z*-average and PDI) but it has been ascertained that it was only a superficial effect because *Z*-potential kept unchanged, as well as the encapsulation efficiency. After the same period of storage, the 0.01% w/v coated D3-loaded liposomes and the 0.005% w/v coated K2-nanoliposomes assured better stability than the uncoated structures. Coated nanoliposomes morphology and all other properties (*i.e.* numerical size, *Z*-average, *Z*-potential, PDI, encapsulation efficiency and mucoadhesiveness), indeed, remained unchanged with respect to freshly prepared ones.

At last, it can be concluded that by the novel developed technique and the optimized chitosan coverage, very stable and mucoadhesive polymer–lipid hybrid nanoparticles, encapsulating vitamin D3 and vitamin K2, are produced as micronutrients delivery systems for potential nutraceutical applications.

## Conflicts of interest

There are no conflicts to declare.

## Supplementary Material
